# Natural Products for Liver Cancer Treatment: From Traditional Medicine to Modern Drug Discovery

**DOI:** 10.3390/nu14204252

**Published:** 2022-10-12

**Authors:** Da Bin Kim, Do Kyeong Lee, Chunhoo Cheon, Rosy Iara Maciel A. Ribeiro, Bonglee Kim

**Affiliations:** 1 College of Korean Medicine, Kyung Hee University, Kyungheedae-ro 26 Dongdaemun-gu, Seoul 02447, Korea; 2 Laboratory of Experimental Pathology, Federal University of São João del Rei—CCO/UFSJ, Divinópolis 35501-296, Brazil

**Keywords:** liver cancer, hepatic cancer, natural compounds, natural products, anticancer, apoptosis, anti-metastasis, antiangiogenesis

## Abstract

Primary liver cancer was the seventh most diagnosed cancer and the second leading cause of cancer death with about 906,000 cases and 830,000 deaths, respectively, in 2020. Conventional treatment for liver cancer, such as transarterial chemoembolization (TACE) or sorafenib, has limitations in that there is the recurrence of cancer, drug inefficacy, and adverse effects. Traditional medicine and natural products of several regions including Korea, China, Europe, North America, India, and the Middle East have attracted a lot of attention since they have been reported to have anticancer effects with low adverse effects. In this review, several in vivo studies on the effects of natural compounds on liver cancer and clinical trials approving their therapeutic benefits were selected and discussed. As a result of the analysis of these studies, the effects of natural compounds were classified into a few mechanisms: apoptosis, anti-metastasis, and antiangiogenesis. In addition, medications including natural products in clinical trials were observed to exhibit improvements in various liver cancer symptoms and patients’ survival rates. This study presents findings suggestive of the anticancer potential of natural products and their properties in relieving related symptoms.

## 1. Introduction

With approximately 906,000 new cases and 830,000 deaths in 2020 worldwide, primary liver cancer was the seventh most diagnosed cancer and the second leading cause of cancer death [1]. Hepatocellular carcinoma (HCC) (75–85% of cases) and intrahepatic cholangiocarcinoma (10–15%), as well as other types, are the most common types of primary liver cancer [1]. In mainstream medicine, several approaches are primarily used in the treatment of liver cancer, such as surgical resection, liver transplantation, radiofrequency, chemotherapy, and targeted molecular therapy. However, disease recurrence and drug toxicity or ineffectiveness, which negatively influence the patient’s quality of life after therapies, remain major issues. For instance, the repeated use of transarterial chemoembolization (TACE) has been related to fibrosis, toxicity, and hypofunction of the liver [2]. Additionally, sorafenib, which is known as the first line therapy for advanced HCC, causes side effects such as hypertension, hemorrhage, neuropathy, leukopenia, lymphopenia, diarrhea, nausea, vomiting, and dyspnea [3].

Natural products found in dietary fruits, vegetables, and spices inhibit mechanisms of cancer development and stimulate mechanisms related to disease prevention by activating antitumor, antiproliferative, antiinflammatory, and antioxidant systems [4,5]. As to cancer therapy, properties of natural products to selectively regulate enzyme activities leading to carcinogenesis are very important. Certain classes of natural products such as alkaloids, phenols, fatty acids, quinones, etc., are considered to be selective inhibitors of the catalytic activity of topoisomerase I/II, which is responsible for the modification of DNA, the cell cycle, and the biology of tumor cells [6].

However, natural products have several drawbacks and require further investigation with sufficient evidence. Despite the demonstration that natural products are structurally optimized by evolution to provide biological functions, there is the potential for adverse effects with natural products themselves [7]. The safety of natural products is often understudied and overestimated due to supplement marketing [8]. In addition, screening sufficient biological material in natural products to isolate and find the properties may also be challenging [9]. Considering the limitations of existing treatments, the potential of natural compounds, studies of their therapeutic effect, and the safety of natural products should be discussed.

In this review, natural compounds found in animals, plants, and fungi possessing curative effects on liver cancer and their mechanisms were presented. Results from in vivo studies and human clinical trials were reviewed. 

## 2. Methods

Articles involving liver cancer and natural compounds from 2016 to 2021 found in “Pubmed”, “Web of Science”, and “Scopus” were selected. The keyword combinations: “liver cancer AND natural compounds” and “hepatic cancer AND natural compounds” were used to find the articles. Inclusion criteria were as follows: (1) studies with in vivo animal experiments that showed anticancer efficacy of natural compounds on liver cancer, (2) clinical trials demonstrating curative benefits related with hepatic cancer, (3) original written research articles in English. The exclusion criteria were (1) review studies, (2) meta-analysis studies. 

## 3. Results

### 3.1. In Vivo Mice Studies—Mixtures

Twenty-five in vivo mice studies have reported the efficacy of natural products in inhibiting liver tumor growth. Among them, seven studies used extracts or mixtures (Table 1).

Chen et al. reported that bear bile powder exerts anticancer activities by inhibiting the STAT3 signaling pathway [10]. Bear bile powder (BBP) was administered to HCC xenograft mice. BBP induced cell apoptosis, and suppressed cell proliferation and vessel density in HCC tumor tissues. In addition, BBP remarkably suppressed the STAT3 phosphorylation and modulated critical target genes such as Bcl-2, Cyclin D1, CDK4, and VEGF-A. 

Caroline Wasonga and Charles Omwandho reported that *Agaricus bisporus* extracts could inhibit the progression of the carcinogenesis [11]. Single intraperitoneal injections of diethylnitrosoamine were given to CBA mice to induce hepatocellular carcinogenesis before the treatment. Compared to the control group, the *Agaricus bisporus* extract-treated mice group showed decreased levels of sialic acid, implying tumor regression. 

Lee et al. reported that a low-carbohydrate diet (LCD) and AMPK-activating phytochemical extracts including green tea, curcumin, quercetin, and resveratrol induced synergic tumor suppression [12]. In this experiment, B16F10 melanoma cells were injected subcutaneously into C57BL6 mice and 5 mg/g of AMPK-activating agents were added to a low-carbohydrate diet formula. Compared with the control group, LCD with multiple phytochemicals suppressed NF-κB and the IGF-1R/PI3K/Akt/mTOR signaling and activated the AMPK/SIRT1/LKB1pathway. 

He et al. described the antitumor effects of Neem tree extract (NTE), which originated from *Azadirachta indica* [13]. In this experiment, H22 cells were injected subcutaneously in Kunming mice to induce HCC, and the mice were treated daily with NTE by intragastric administration. Such effects can be explained by NTE improving the non-specific immune function of H22-bearing mice. 

Qiu et al. reported that Rhizoma Paridis saponins (RPS) extract, which originated from *Paris polyphylla* var. *yunnanensis* could suppress tumor growth [14]. Administration of this natural product decreased tumor weight by downregulating the serum levels of lactate, acetate, N-acetyl amino acid, and glutamine signals. According to the analysis, RPS activated tumor suppressor p53 and PTEN, and suppressed FASN to inhibit lipogenesis. In addition, it repressed Myc and GLS expression and decreased the glutamine level.

Sun et al. elucidated the antitumor effect and mechanism of total saponins, isolated from *Schizocapsa plantaginea*, which is well known for its therapeutic properties in traditional Chinese medicine [15]. The SMMC-7721 cells were injected into the nude mice, and the mice were treated with the extract. It was found that tumor volume and weight decreased. The activation of JNK and Erk1/2 and inactivation of p38MAPK were observed after the treatment. 

Tomato powder (TP) is shown to prevent high-fat diet (HFD)-induced inflammation and inhibit HFD-promoted hepatic cancer development by modulating gut microbiota [16]. In this experiment, BCO1−/−BCO2−/− double knockout mice were fed an HFD with TP. An increase in sirtuin 1 activity, nicotinamide phosphoribosyltransferase expression, and NAD^+^ level, and a decrease in IL1β, IL6, IL12α, and MCP1 were observed after the treatment. 

### 3.2. In Vivo Mice Studies—Single Compounds

Sixteen studies reported the therapeutic effects of single compounds on liver tumors (Table 2). They are classified into several chemical categories: alkaloids, fatty acids, hydrocarbons, polyphenols, polysaccharides, and quinones. 

#### 3.2.1. Alkaloids

Berberine is an iso-quinoline alkaloid derived from the Berberis species, and it is famous for its anticancer, antiinflammation, and antibacterial activities [17]. Zheng et al. investigated the antitumor effect of berberine in ICR mice liver tumor xenograft model. In this study, the researchers developed a proof-of-principle of a facile strategy to produce carrier-free fluorescent berberine microrods (Ber-MRs) in gram scale through an antisolvent precipitation method. Administration of this natural product resulted in a decrease in the size and weight of the tumor, without any significant toxicity. 

Britanin is a sesquiterpene lactone compound that was reported to have antitumor effects on HCC [18]. The low-dose, high-dose groups of mice were injected with a britanin dose extracted from *Inula linariifolia Turcz* and in vivo bioluminescence imaging was conducted. The tumor size measurements indicated britanin treatment inhibited tumor growth by upregulating the expression of Bax and downregulating the expression of Bcl-2, and suppressing NF-κB activation, which is related to a decrease in nuclear p65 levels.

#### 3.2.2. Fatty Acids

Wang et al. reported that doxorubicin-loaded docosahexaenoic acid nanoparticles (DOX-nano) significantly inhibited the tumor growth [19]. HepG2/DOX cells were injected into BALB/c mice, and after that, the mice were injected with DOX-nano at 2 mg/kg for 10 days. In the DOX-nano group, the weight of the tumor tissues were significantly lighter, and the expression of multidrug resistance-related proteins, including MRP, LRP, BCRP, Bcl-2, and PKC-α was significantly lower.

Ou et al. reported that low-density lipoprotein docosahexaenoic acid (LDL–DHA) nanoparticles induced ferroptotic cell death in a HepG2 liver cancer xenograft BALB/C mice model [20]. The administration of LDL–DHA at a dose of 2.5 mg/kg for three consecutive days inhibited the tumor growth by increasing lipid hydroperoxides and downregulating GPx-4.

#### 3.2.3. Hydrocarbons

Atractylon is a sesquiterpenoid isolated from *Atractylodes lancea* and *Atractylodes chinensis* [21]. Cheng et al. elucidated that atractylon inhibited the hepatic cancer growth and the EMT process. In this experiment, the NOD/SCID mice were implanted subcutaneously with HepG2 cell suspensions into the left dorsal. After that, the atractylon was dissolved in corn oil and given to the mice by intragastric administration. Compared to the control group, the increase in E-cadherin, TIMP2, Bax, and cleaved caspase-3 and the decrease in Ki-67, N-cadherin, Vimentin, α-SMA, MMP-2, MMP-9, and Bcl-2 were observed in the atractylon-treated mice group. 

Liu et al. described the antitumor effects of GL22, which originated from *Ganoderma leucocontextum* [22]. The *Ganoderma* species of mushroom is a well-known traditional Chinese medicine, widely used to treat and prevent cancer. In this experiment, Huh7.5 cells were subcutaneously injected into BALB/C nu-nu mice. The administration of this natural product inhibited tumor growth by downregulating PPARα, PPARγ, FABP1, FABP4, and FABP5. 

Rotundic acid (RA) exhibited anticancer activity and the inhibitory effect of tumor growth in BALB/c nude mice [23]. RA was extracted from the barks of Ilex. rotunda, commonly known as “Jiu Bi Ying”, which in China is used as a traditional Chinese medicine. Administrations of RA reduced HepG2 tumor weights and volumes in a mouse model. This demonstrated that RA treatment induced apoptosis, with a notable enhancement of the expression levels of phosphor-p38 MAPK, which is known to induce cell death and suppress angiogenesis. Additionally, downregulation of proliferation marker Ki-67, angiogenesis marker CD-3, and AKT/mTOR pathway proteins appeared as a result of the treatment.

Ursolic acid (UA) is a natural triterpenoid compound found in plants, herbs, and other kinds of food [24]. It is known to have antiinflammatory, hepatoprotective, anti-allergic, and anticancer effects. UA suppressed tumor growth and STAT3 phosphorylation in a HepG2 liver cancer xenograft mice model. The level of p-STAT3 and Bcl-2 were decreased, and the cleavage of caspase-3 was upregulated in the UA group. 

Wani et al. described the therapeutic effects of zerumbone, which was isolated from *Zingiber zerumbet* [25]. In this experiment, Huh7 cells were injected in NSG mice to induce HCC, and the mice were treated daily with zerumbone by intraperitoneal injection. The tumor size, weight, and volume were significantly lower, and subcutaneous and orthotopic tumor growth were suppressed in a zerumbone-treated mice group. 

#### 3.2.4. Polyphenols

Yao et al. reported that 747 from *Abies georgei*, which is known as a natural C-C chemokine receptor type 2 (CCR2) antagonist, enhanced the tumor immunosuppressive microenvironment and the therapeutic effect of sorafenib [26]. CCR2 is a feature of inflammatory monocytes, and the infiltration of various immune cells is responsible for HCC initiation and progression. Hepa1–6 or LPC-H12 cells were injected into the BALB/c athymic nude mice and C57BL/6 mice to induce HCC. The administration of 747 suppressed liver cancer growth, reduced the infiltration of tumor-associated macrophages, and increased CD8 T cells. Moreover, 747 enhanced the antitumor effects of sorafenib by improving the quantity and distribution of CD8 T cells within tumors.

Xiao et al. showed the anticancer effect of biochanin A with Western blot analysis [27]. They treated HCC xenograft nude mice with biochanin A found in red clover, chickpeas, and several other plant sources. The Western blot of xenograft mice indicated the inhibitory effect of biochanin A on the expression of proliferating cell nuclear antigen (PCNA) and the activation of the ERK, MAPK, and PI3K/AKT/mTOR signaling pathways. 

Iscador P, mistletoe extract, includes viscumins, which are highly cytotoxic to tumor cells [28]. In this experiment, Iscador P was given to female mice as a single injection. As a result, Iscador P decreased the activity of lysosomal hydrolases and increased aminopeptidases and β-D-glucuronidase in the cytosol. Moreover, hepatocyte mitochondria were enlarged and increased due to the inhibition of protein synthesis leading to autophagy, which is a promising therapeutic strategy for anticancer treatment.

Shi et al. reported that melanin of *Lachnum* (LM) and its derivative (ALM) improved the immune functions and inhibited angiogenesis [29]. In this experiment, H22 cells were injected into the Kunming mice to induce liver cancer. The administration of LM and ALM resulted in a decrease in the weight and volume of the tumor. Moreover, serum concentration of IL-2, IL-6, TNF-α, and interferon gamma (IFN-γ) were increased and VEGF and bFGF were decreased. 

Li et al. demonstrated in vivo the anticancer potential of psoralidin [30]. Each group of xenografted mice were injected with HepG2 cells subcutaneously at the flank and injected with psoralidin from Psoralea corylifolia. Psoralidin induced a reduction in the tumor volume and weight. Moreover, it was observed that psoralidin upregulated Bax, downregulated Bcl-2, and triggered autophagy, apoptosis, and cell cycle arrest in the HepG2 cells, which leads to the suppression of tumor growth. 

Xiao et al. reported that TTF1-NP (5,2′,4′-trihydroxy-6,7,5′-trimethoxyflavone), which was isolated from *Sorbaria sorbifolia* could suppress tumor growth [31]. *Sorbaria sorbifolia*, a medicinal plant that lives near Chang Bai mountain in China, has been traditionally used to treat cancer. In this experiment, HepG2 cells were implanted in BALB/c nude mice. Compared to the vehicle group, a TTF1-NP-treated mice group showed a smaller tumor size and reduced STAT3 and p-STAT3 protein expression. 

#### 3.2.5. Polysaccharides

*Acanthopanax senticosus* (ASPS) was reported to have immunomodulatory activities [32]. Polysaccharide extracted from ASPS was observed to have an inhibitory effect on the growth of a hepatic carcinoma H22 cell in solid tumor-bearing mice. High and medium doses of ASPS significantly increased serum IL-2 and IL-12 levels in H22 solid tumor-bearing mice. Additionally, treatment with high, medium, and low doses of ASPS significantly increased INF-γ expression in H22 solid tumor-bearing mice compared with the control group. 

Fucoidan, natural polysaccharides extracted from brown algae, contributed to the inhibition of the motility and proliferation of HCC cells in vivo [33]. “HCC-afflicted mice” were divided into the fucoidan group and the control group, and tumor weight and volume in the fucoidan group were significantly decreased. Its inhibitory effect is attributed to downregulation of the micro RNA522 gene and upregulation of Long Inergenic Non-Protein Coding RNA 261 (LINC00261), which plays a role as a regulator of tumor suppressor genes. 

#### 3.2.6. Quinones

Shikonin is a multifunctional bioactive natural product isolated from *Lithospermum erythrorhizon* [34]. Song et al. reported that the combination of shikonin and arsenic trioxide (ATO) exhibited synergistic anticancer efficacy and achieved greater selectivity between cancer cells and normal cells. The administration of shikonin significantly enhanced the anticancer effect of ATO by upregulating caspase 3, caspase 9 activities, and the levels of CHOP mRNA, and MDA in tumor tissues. 

A total of 25 studies have been performed to test the antitumor activity of natural products using mice (Table 1, 2). Twenty studies focused on plant-derived compounds, two studies on fungi-derived compounds [11], and only one study presented a natural substance from animal origin [10]. Most studies using mice were performed on Kunming (7), BALB/C (6), and ICR (3) mice, and were confirmed by mechanisms such as STAT3, caspase, Bcl-2, Bax, IL, and PPAR. As well as inhibitory effects on tumor growth, some natural compounds showed the induction of cell apoptosis [10,18,23,30]. Some studies explained their efficacy through regulations of genes such as Bcl-2, Bax, and LINC00261 [18,30,33], and other studies presented antitumor mechanisms involving changes in enzyme expression [27,28]. Some reports lacked the source of the compound [12,16,17,24,27,33]. In several studies, the experimental models were not described [10,15,16,24,30]. Meanwhile, some experiments lacked detailed mechanisms [13,17,35,36,37,38]. However, several studies were designed elaborately by highlighting both in vivo and in vitro experiments [18,23,27,33]. 

### 3.3. In Vivo Rat Studies—Mixtures

Twelve studies reported the efficacy of natural products through in vivo rat examination (Table 3). Among them, five studies demonstrated efficacies of the extracts from natural compounds. 

Daily doses of Nigella sativa extract administered to rats exhibited chemopreventive effects on diethylnitrosamine (DENA)-induced hepatocellular carcinoma [39]. *Nigella sativa* extract protected the liver by inhibiting EGFR/ERK1/2 signaling caused by DENA that led to upregulation of the expression of PCNA, c-fos, Bcl2, and cell proliferation. Moreover, high antioxidant activity of *Nigella sativa* supported the inhibition of carcinogenesis. 

Khan et al. demonstrated the therapeutic effects of Ajwa dates extract (ADE), which originated from *Phoenix dactylifera* [36]. Date fruit is a native fruit of the Middle East, and it is unique for its medicinal properties. In this experiment, diethylnitrosamine (DEN) was used to induce hepatic cancer in Wistar rats. The ADE-treated group showed the reversal of DEN-damaged liver towards normal, provoked by the increase in SOD, GR, GPx, ALT, AST, ALP, IL-2, and IL-12 and decrease in IL-1α, IL-1β, GM-CSF, AFP, and IL-6. 

Jianpi Huayu Decoction (JHD) is a widely used traditional Chinese herbal formula [40]. JHD extract suppressed tumor growth and epithelial–mesenchymal transition in Wistar rats. The expression levels of E-cadherin and EpCAM were increased, and the levels of N-cadherin and vimentin were decreased in the JHD treatment group. 

Ahmed et al. conducted a study of the antitumor effects of *Hemimycale arabica* and *Negombata magnifica* mesohyls from marine sponges in HCC rat models [41]. HCC-afflicted rats treated intraperitoneally with *Hemimycale arabica* and *Negombata magnifica mesohyls* had lower levels of AST, ALT, total bilirubin, direct bilirubin, indirect bilirubin, and GGT, which indicated their hepatoprotective potency. Levels of liver cancer biomarkers such as alpha fetoprotein (AFP), alpha-fucosidase (AFU), and carcinoembryonic antigen (CEA), which are significantly increased by HCC, were depleted in the *Hemimycale arabica* and *Negombata magnifica* mesohyls groups. 

Defatted sticky rice bran extract, especially purple rice bran extract (PRBE), was reported to have protective effects on the early stages of hepatocarcinogenesis in rats [42]. PRBE decreased the number and size of the hepatic glutathione S-transferase placental form (GST-P) positive foci in rat hepatocarcinoma initiated with DEN. PRBE also downregulated the expression of TNF-α, iNOS, and NF-κB and anthocyanins and other phenolic compounds in PRBE, which inhibited proliferation and inflammation. 

### 3.4. In Vivo Rat Studies—Single compounds

Seven studies were on single compounds, and their classifications are fatty acids, hydrocarbons, polysaccharides, polyphenols, and quinones (Table 4). 

#### 3.4.1. Fatty Acids

Wen et al. reported that low-density lipoprotein docosahexaenoic acid (LDL–DHA) nanoparticles inhibited redox reactions in tumor tissues [43]. In this experiment, H4IIE cancer cells were implanted into AxC-Irish rats. The administration of LDL–DHA decreased the level of GPx-4. 

#### 3.4.2. Hydrocarbons

You et al. reported that phyllanthin from *Phyllanthus* species has significant anticancer activity against the DEN-induced liver cancer in rats [44]. The administration of phyllanthin inhibited DEN-induced liver damage through caspase-dependent apoptosis via the upregulation of p53 and Bax, and the downregulation of Bcl-2, which mediates the mTOR/Akt signaling pathway and leads to the suppression of tumor initiation and progression. 

Zerumbone from *Zingiber zerumbet* was reported to have in vivo antiproliferative and antiangiogenic properties [38]. Zerumbone-treated rats with DEN-induced HCC had lower median liver weight than those non-treated. Moreover, zerumbone treatment stimulated cell apoptosis and downregulated the expression of VEGF and MMP-9, which are mediators of angiogenesis.

#### 3.4.3. Polysaccharides

Arabinoxylan from rice bran administered to rats exhibited protection against chemically-induced carcinogenesis and apoptosis via the mitochondrial pathway [45]. Arabinoxylan alleviated liver preneoplastic lesions and induced the arrest of the cancer cell cycle in the sub-G1 phase. It also downregulated the expression of Bcl-2 and upregulated p53, Bax, and caspase-3. Moreover, it inhibited the suppression of NF-κB/p65 gene expression caused by cancer. 

#### 3.4.4. Polyphenols

Sadeeshkumar et al. reported that dieckol, which is a major polyphenol of marine brown seaweed *Ecklonia cava*, regulates apoptosis, inflammation, invasion, and angiogenesis [46]. In this experiment, N-Nitrosodiethylamine (NDEA) was injected into Wistar rats to induce hepatocarcinogenesis. The administration of dieckol downregulated the expression of Bcl-2, caspases MMP2/9, VEGF, NF- κB, and COX-2, and increased the expression of Bax, cytochrome c, and caspase-3.

Krishnan et al. reported that hesperetin (5,7,3′-trihydroxy-4′methoxy flavanone), which is abundant in citrus fruits, inhibited cell inflammation and proliferation [47]. In this experiment, DEN was used to induce hepatocarcinogenesis in male Wistar albino rats. The administration of hesperetin, intraperitoneally, decreased the number of mast cells, TNF-α, NF-κB, glycoconjugates, PCNA, and argyrophilic nucleolar organizing regions.

#### 3.4.5. Quinones

Coenzyme Q 10 treatment at a dose of 0.4mg/kg thrice a week for 2 weeks inhibited cell proliferation and histological alteration of liver [48]. Coenzyme Q 10 treatment increased lipids and cleaved caspase 3 and decreased TNF-α, AFP, Bcl-2, SRB1, phospholipase D (PLD) activity, and CD59. Most of all, increased lipids and a reduction in the expression of CD59 and PLD activity indicated that Coenzyme Q 10 treatment has an important role in protecting the liver against rat HCC. 

All 12 studies were conducted by using sources presenting inhibitory efficacies on tumor growth and some of them included cell cycle arrest [45], liver protection [41,44], and apoptosis [38,41,45,46]. Most studies using rats were performed on Wistar rats and were confirmed by mechanisms such as SOD (3), ALP (3), c-caspase-3 (3) and NF-κB (3), p53, and Bax (2). Some reports lacked the source of the compound [42,43,44,47,48], and some studies lacked the classifications of the compound [43]. Meanwhile, some other studies presented both in vivo and in vitro experiments [41,44,49]. Moreover, toxicity tests toward normal hepatic cells confirmed the safety of the compounds in certain studies [36,39]. The study using coenzyme Q 10 [48] is significant in that it is the first to explain an anticancer mechanism through the reduction of CD59 and PLD. The chemical structures of the single compounds are listed in Table 5. 

### 3.5. Clinical Trials

Eight studies reported the efficacy of natural products through clinical trials (Table 6).

Fuzheng Jiedu Xiaoji formulation (FZJDXJ) is the most prescribed traditional Chinese herbal medicine for HCC treatment in Beijing [50]. FZJDXJ consists of a variety of herbal medicines, including *Codonopsis pilosula, Astragalus mongolicus, Atractylodes macrocephala, Angelica sinensis, Poria cocos, Adenophora stricta, Ophiopogon japonicus, Rehmannia glutinosa, Paris polyphylla, Curcuma phaeocaulis,* and *Pinellia ternate.* Compared to the standard treatment group, FZJDXJ combined with TACE therapy significantly prolonged one-year overall survival (OS) and progression-free survival (PFS) and reduced the mortality rate of HCC patients. A total of 1619 active constituents of FZJDXJ, including *formononetin,* and *chlorogenic acid* were identified by HPLC-MS/MS. These data suggested that FZJDXJ effectively inhibited the proliferation and migration of liver cancer cells through the modulation of the AKT/CyclinD1/p21/p27 pathways. 

Jiedu Granule is a traditional Chinese medicine composed of *Salvia chinensis*, *Actinidia valvata*, *Gallus gallus domesticus*, and *Cremastra appendiculata* [51]. Wang et al. elucidated that Jiedu Granule combined with TACE plus gamma knife radiosurgery (GKR) is safe in HCC patients with portal vein tumor thrombosis (PVTT). Compared to the single TACE plus GKR group, the Jiedu granule combined group showed longer median OS. The median OS rates in the single TACE plus GKR and Jiedu granule combined group were 11.3 months and 15.8 months, respectively.

Changou et al. performed a phase II safety and efficacy clinical trial of PHY906 and capecitabine in patients with advanced HCC [52]. PHY906 is a pharmaceutical-grade formulation of four traditional Chinese herbs, including *Scutelleria baicalensis*, *Paeonia lactiflora*, *Glycyrrhiza uralensis,* and *Ziziphus jujuba.* In this experiment, the median progression-free survival was 1.5 months, and median OS was 6 months with a 51.3% 6-month survival rate. The data showed that PHY906 increased the therapeutic index of capecitabine by enhancing its antitumor activity and reducing its toxicity profile in advanced HCC.

Zhai et al. reported that traditional herbal medicine (THM) treatment, which is a mixed aqueous extract of *Bufo gargarizans*, *Actinidia valvata*, *Salvia chinensis*, *Pseudobulbus cremastrae*, and *Gallus gallus domesticus*, was superior to that of TACE in preventing recurrence in patients with small HCC [53]. Recurrence-free survival (RFS) in the THM group was significantly higher than that in the TACE group. Moreover, OS in the THM group was found to be significantly superior to that in the TACE group.

TACE combined with Huaier granule was reported to treat primary hepatic cancer safely and effectively [54]. Patients randomly assigned to an experimental group received TACE with Huaier granule showed higher 6-month and 1-year tumor response rates than those of a control group, which received chemotherapy only. Moreover, OS at 12 months was found to be better in the experimental group than in the control group. 

The decoction of Jian Pi Li Qi (JPLQ), a traditional Chinese medicine, has been shown to relieve symptoms of poor quality of life of patients in HCC following TACE [55]. In total, 140 patients were randomly allocated to one of three groups, A (patients receiving neither herbal medicine nor placebo), B (placebo group), or C (JPLQ group), and their QOL score, as well as liver function, were evaluated. Symptoms of postembolization syndrome including fever, pain, fatigue, nausea, disturbed sleep, distress, lack of appetite, drowsiness, dry mouth, vomiting, constipation, and bloating increased harshly after TACE and were significantly alleviated by JPLQ. In addition, the total bilirubin level in group C was lower than in A and B. 

Ye et al. conducted a multicenter, randomized, double-blind, placebo-controlled trial of Shuangbai san [56]. Among 134 patients with mild cancer related to liver cancer, the group receiving Shuangbai san showed a significantly more decreased NRS (numerical rating scale) score of pain than that of the control group receiving a placebo. Moreover, QOL scales for patients in the Shuangbai san group were greater than in the control group, indicating Shuangbai san can relieve various symptoms of liver cancer such as abdominal distention, diarrhea, anorexia, nausea, and dizziness. 

Chay et al. reported that patients who received Coriolus versicolor (CV) had significantly higher physical, cognitive, and emotional functioning scores and less appetite loss and scores of symptoms including nausea, vomiting, pain, insomnia, constipation, and diarrhea than those of a control group [54,57]. Progression-free survival and OS were both longer in the CV group than in the placebo group. Moreover, the use of CV was reported to stabilize interleukin-17F, prolactin, and TNP-related apoptosis-inducing ligand R1 levels.

All eight studies proved the efficacies of the medications targeting the symptoms of liver cancer. Among them, one study included a phase of the trial [52,58]. Some studies performed randomized, double-blind, placebo-controlled trials [50,51,55,56], and one of them conducted the trial in multiple centers [56]. Only one study was a phase II clinical trial [52]. Three studies lacked a double-blind assignment and recruited fewer patients (39, 62, 6, 15, respectively) [52,54,57,58]. One study lacked sources of the medications [54,58]. Although a total of eight studies confirmed the effectiveness of their treatments, they all had a limited number of patients, which requires further exploration with a larger population.

## 4. Discussion

Liver cancer is a common malignant tumor worldwide, mainly associated with chronic viral hepatitis such as hepatitis B or C, alcoholic hepatitis and cirrhosis patients, and liver diseases such as Wilson’s disease and Budd–Chiari syndrome. Even though multiple studies have been undertaken to determine the best treatment approaches, conventional medicines have been linked to various adverse effects. As a result, innovative therapeutic procedures are required, and traditional herbal medicines have attracted a lot of attention in this area for their anticancer activity and synergy with Western medications. Herbs and medications used in traditional Korean medicine also involve the natural compounds that significantly benefit liver cancer patients. For instance, curcumin has been shown to suppress hepatoma cell line (HepG2 cells) proliferation, and total ginsenosides from the roots of *Panax ginseng* mediate and regulate immune responses, thus being able to exhibit anticancer effects [59]. Furthermore, the combination of these two products has been demonstrated to inhibit the PD-1/PD-L1 protein expression leading to synergetic downregulation of the protein expression of PD-L1 and NF-κB, and reduction in tumor growth [59]. Although numerous in-depth investigations have been conducted on the anticancer effects of natural chemicals, further research on the biological functions of herbal medicine in humans is still required. Hence, this study investigated the anticancer effects and associated mechanisms of each natural component and provided a thorough interpretation of the feasibility of employing numerous natural products to treat liver cancer.

Previous studies have explored the relationship between natural products and liver cancer. Man et al. brought together conventional pharmaceuticals and natural compounds that are effective for liver cancer, but the scope of the study was limited to clinical trials [60]. Li et al. investigated the action of various bioactive components of licorice, which is a representative herbal drug used in traditional Chinese medicine [61]. Shan et al. reviewed prior research on the active components in natural products that have been shown to have anti-hepatic fibrosis properties [62]. However, the study’s focus was restricted to cirrhosis, and clinical trials were excluded. In this study, the anticancer efficacy and mechanisms of various natural products that were revealed by in vivo mice and rat experiments as well as clinical trials were summarized. 

Natural compounds that treat liver cancer act by several mechanisms that can be grouped into apoptosis, anti-metastasis, and antiangiogenesis.

### 4.1. Apoptosis

**Figure 1:**
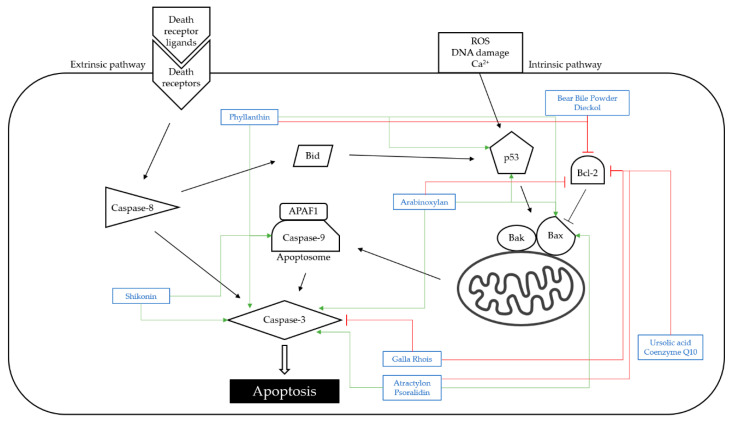
Schematic diagram of apoptosis pathway. The extrinsic pathway starts with covalent bonds of death receptors and ligands, which activate caspase-8 and caspase-3 sequentially to lead to apoptosis. The intrinsic pathway passes through p53 and mitochondria to the apoptosome, which is a combination of APAF 1 and caspase-9 and leads to the cell death. This process is upregulated by the pro-apoptotic proteins such as Bax, Bak, Bid and downregulated by anti-apoptotic proteins such as Bcl-2, Bcl-xL. Caspase-9 was regulated by shikonin, and caspase-3 was controlled by shikonin, phyllanthin, arabinoxylan, Galla Rhois, atractylon, and psoralidin. Bax was upregulated by phyllanthin, atractylon, and psoralidin, and p53 was controlled by phyllanthin and arabinoxylan. Moreover, Bcl-2 was downregulated by bear bile powder, dieckol, phyllanthin, arabinoxylan, Galla Rhois, atractylon, psoralidin, ursolic acid, and coenzyme Q10. The mechanisms of natural products affecting the apoptosis pathway were indicated. The upregulations of the molecules were marked with a green arrow and the downregulations with a red arrow.

Apoptosis is one of the forms of cell death. Apoptosis pathways are generally two types: extrinsic (also known as receptor-mediated) pathways and intrinsic (also known as mitochondria-mediated) pathways (Figure 1). The death receptors are covalently attached to the death receptor ligands in the extrinsic pathway, which causes caspase-8 and caspase-3 to be activated sequentially, cleaving target proteins and leading to apoptosis [63]. The anti-apoptotic proteins, cellular FADD-like IL-1β-converting enzyme-inhibitory protein (c-FLIP) and X-linked inhibitor of apoptosis protein (XIAP), which block caspase-8 and caspase-3 activation, respectively, impede this pathway. Intrinsic death signals, such as reactive oxygen species (ROS), DNA-damaging chemicals, or Ca^2+^ mobilization, initiate the mitochondrial pathway by causing cytochrome c release and the formation of apoptosome, which consists of apoptotic protease activating factor 1 (APAF1) and caspase-9. The tumor suppressor protein p53 mediates this process by directly activating B-cell lymphoma 2-associated X (Bax) and Bak. At the apoptosome, caspase-9 is activated, which then activates pro-caspase-3. Pro-apoptotic proteins such as Bax, Bak, Bid, and second mitochondria-derived activator of caspase (Smac)/direct inhibitor of apoptosis-binding protein with low pI (DIABLO), as well as anti-apoptotic proteins such as B-cell lymphoma 2 (Bcl-2), B-cell lymphoma-extra large (Bcl-xL), myeloid leukemia 1 (Mcl-1), and XIAP, regulate this death process. Caspase-8 causes Bid to be cleaved, causing Bax and Bak to be translocated to the mitochondrial membrane, amplifying the intrinsic apoptosis process.

### 4.2. Metastasis

**Figure 2:**
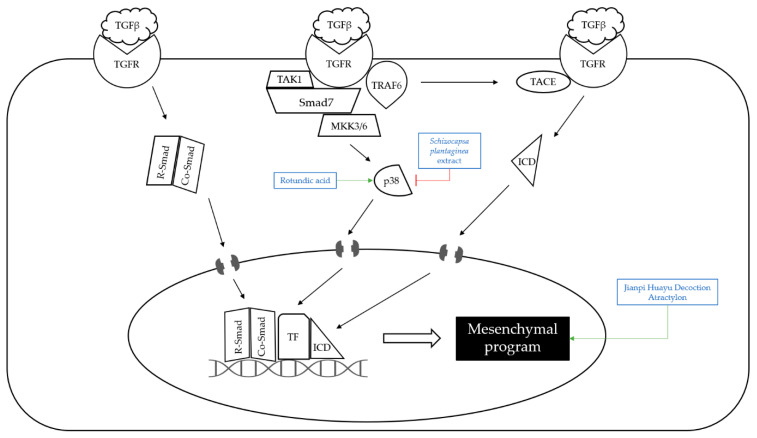
Schematic diagram of metastasis. Metastasis starts with TGF receptor stimulation, which induces the complex of R-Smad and Co-Smad, as well as p38 and ICD to move into the nucleus. These factors control gene transcription to upregulate the mesenchymal program. P38 was upregulated by rotundic acid and downregulated by *Schizocapsa plantaginea* extract. Jianpi Huayu Decoction and atractylon upregulated the mesenchymal program. The mechanisms of natural products affecting metastasis were indicated. The upregulations of the molecules were marked with a green arrow and the downregulations with a red arrow.

Most cancer-related fatalities are caused by metastasis (Figure 2). Tumor invasion and metastasis are the outcomes of a multistep process that includes the proteolysis of the extracellular matrix, which enables abnormal cells to migrate into it. For cancer cells to begin metastasis, the epithelial–mesenchymal transition (EMT) is critical. EMT is defined by the loss of epithelial cell markers, such as E-cadherin, and the increase in mesenchymal markers, such as N-cadherin, vimentin, and α-smooth muscle actin (α-SMA) [40]. Transforming growth factor β (TGF-β) is known as one of the key factors triggering EMT, and Smad transcription factors are part of a pivotal signaling cascade downstream of transforming growth factor (TGF) receptors [64]. There are three different pathways of TGF signaling to induce EMT. First, as a result of TGF receptor (TGFR) stimulation, R-Smad forms a complex with Co-Smad and it binds to DNA with transcription factors. Second, TGF receptor signaling to p38 mitogen-activated protein kinase (MAPK) is mediated by several kinases such as TNF receptor-associated factor 6 (TRAF6), TGF-β-activated kinase 1 (TAK1), mitogen-activated protein kinase 3/6 (MKK3/6), and p38, which rely on Smad7 as a platform. The transcriptional complex formed by Smads and TFs is fed by MAPK signaling. Third, the TGFR–TRAF6 complex triggers the tumor necrosis factor alpha converting enzyme protease, by which TGFR1 is cleaved, and the intracellular domain (ICD) is released. ICD enters the nucleus and attaches to chromatin. The three pathways listed above control gene transcription, resulting in epithelial program downregulation and mesenchymal program upregulation.

### 4.3. Angiogenesis

**Figure 3:**
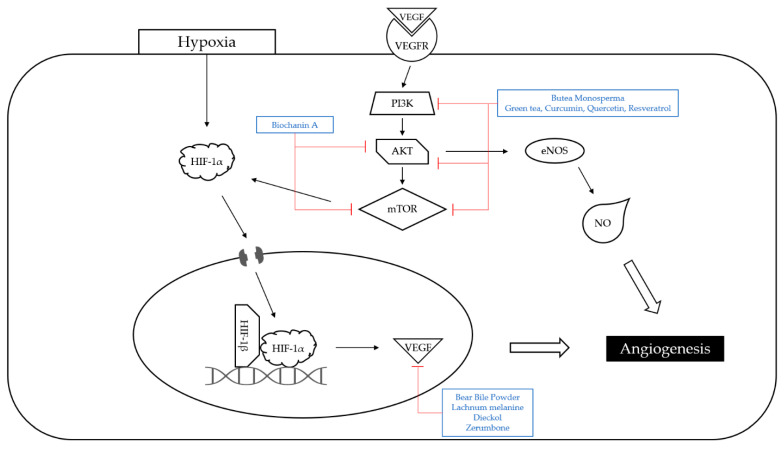
Schematic diagram of angiogenesis pathway. HIF-1α, which is activated by hypoxia, induces VEGF synthesis and leads to angiogenesis. Moreover, PI3K/AKT/mTOR pathway regulates cell proliferation. PI3K was downregulated by *Butea Monosperma* and mixture of green tea, curcumin, quercetin, resveratrol extracts. Both AKT and mTOR were inhibited by Biochanin A, *Butea Monosperma* and mixture of green tea, curcumin, quercetin, resveratrol extracts. VEGF was downregulated by bear bile powder, Lachnum melanin, dieckol, and zerumbone. The mechanisms of natural products affecting angiogenesis were indicated. The upregulations of the molecules were marked with a green arrow and the downregulations with a red arrow.

Angiogenesis, a series of processes in which new blood vessels are formed from existing blood vessels, is critical for tumor growth and survival (Figure 3). In the recent decade, antiangiogenic therapy has emerged as a key field of study in cancer treatment. Angiogenesis is an essential process to maintain homeostasis as part of normal physiological action. It is generally accompanied by and driven by excessive production of angiogenic agents and decreased expression of angiogenesis inhibitors. Hypoxia, a frequent symptom of solid tumors, greatly activates vascular endothelial growth factor (VEGF), a powerful pro-angiogenic agent that is critical for tumor vascular expansion [65]. Although VEGFs are direct ligands for endothelial cells, numerous other growth factors generated by cancer cells as well as activated endothelial cells stimulate angiogenesis, with many of them upregulating VEGF synthesis. Other angiogenic factors including nitric oxide and angiopoietins are also influenced by the phosphatidylinositol 3-kinase (PI3K)/protein kinase B (AKT) pathway [66]. PI3K are enzymes involved in phosphorylation of phosphatidylinositol. Phosphatidylinositol 3,4,5-triphosphate (PIP3), which is a lipid messenger produced by PI3K, activates AKT. AKT leads to the phosphorylation of the mammalian target of rapamycin (mTOR), which regulates cell proliferation and protein production. When the PI3K/AKT pathway is active in tumor cells, pathways associated with hypoxia-inducible factor 1 (HIF-1) can enhance VEGF synthesis. 

### 4.4. Limitations and Prospects of This Study

There were several limitations of this study. The time limit for conducting literature searches was established at five years, and articles written in non-English were excluded. Some studies omitted basic information such as classification, source, experimental model, and mechanism. Due to the nature of the study on multitarget medicine, human studies were scarce, and mechanical research was also insufficient.

Further studies on herb-induced liver injury (HILI) and the safety of the related natural products are needed for the evidence of this study. Herbal medicine may also have adverse effects, such as hepatotoxicity, nephrotoxicity, cardiotoxicity, neurotoxicity, and carcinogenicity [67]. Among these effects, hepatotoxicity is the most common side effect and HILI accounts for about 25% of all drug-induced liver injury [68]. Difficulties ranging from asymptomatic or abnormal hepatic biochemical tests to acute liver failure requiring a liver transplant can be represented as clinical manifestations of HILI [69]. For instance, Polygonum multiflorum Thunb. (PM) is the most common herb that can lead to HILI, with a hepatocellular pattern of injury at presentation, and higher transaminase and bilirubin levels than conventional DILI cases. The immune-promoting components in PM are considered to make the liver vulnerable to certain components, which activates the overexpression of inflammatory cytokines, leading to liver injury [70]. Although most of the experiments and trials reviewed in our study did not present significant hepatotoxicity and adverse effects shown by natural compounds, the awareness about the harmfulness of certain natural products such as PM is important. Therefore, further validation of the usage of natural products based on risk assessment is required. 

Furthermore, this review lacks the confirmation process of whether the studies listed had statistically significant effects. The inclusion criteria of this review did not include “research that demonstrated reliable statistical analysis data (*p*-values that were less than 0.05)”. This is because this study focused on providing a broader perspective by targeting as many natural products as possible that are effective in liver cancer without being bound by statistical analysis. 

Research on the anticancer effects of natural products is valuable because they have the potential to create synergy with conventional drugs. Simultaneously, as single-targeted conventional medicines have demonstrated their limits, multitarget herbal medicines have sparked increased interest in hepatic cancer. Thus far, it has been difficult and time-consuming to figure out the interactions of multitarget extracts. Therefore, further studies into the synergistic impact of conventional medications and natural products for hepatic cancer is urgently needed. By exploring the therapeutic effects and mechanisms of traditional herbal medicine, researchers would be able to better understand how multiple compounds have large cytotoxic effects on target cancer cells, allowing them to have a broader perspective in clinical circumstances.

## 5. Conclusions

This study examined the natural products that showed antitumor activity against hepatic cancer, providing an overview that clarified their effectiveness and the mechanisms by which they work. The natural products have proven to be an important source of treatment and prevention for liver cancer, with their effects working through several mechanisms. The results of this study are expected to lay the scientific groundwork for the creation of novel medications that may provide a broader perspective in clinical treatment. Further studies into the synergistic impact of conventional medications and natural products for liver cancer are needed.

## Figures and Tables

**Table 1:** In vivo mice—mixtures.

Classification	Compound/Extract	Source	Experimental Model	Dose; Duration	Efficacy	Mechanism	Reference
Animal	Bear Bile Powder	*Fel Ursi*		10 mg/kg; 3 weeks	Induction of cell apoptosis Inhibition of tumor growth	↓Bcl-2, Cyclin D1, CDK4, VEGF-A ↑Bax	[10]
Fungi	*Agaricus* *Bisporus* extracts	*Agaricus* *bisporus*	CBA mice	1 g; 12 weeks	Inhibition of the progression of the carcinogenesis	↓Sialic acid	[11]
Plant	Green tea, curcumin, quercetin, resveratrol extracts		C57BL6 mice	5 mg/g; 50 days	Increase in the survival time	↑AMPK, SIRT1, LKB1 ↓ IGF-1R, PI3K, AKT, mTOR, NF-κB	[12]
Plant	Neem tree extract	*Azadirachta indica*	Kunming mice	150, 300, 600 mg/kg; 27 days	Increase in survival rate Inhibition of H22 tumor growth		[13]
Plant	Rhizoma Paridis saponins extract	*Paris polyphylla* var. *yunnanensis*	Kunming mice	100 mg/kg; 14 days	Decrease in tumor weight	↓Lactate, acetate, N-acetyl amino acid	[14]
Plant	*Schizocapsa plantaginea* extract	*Schizocapsa plantaginea*		10, 25, 50 mg/kg; 15 days	Inhibition of tumor growth	↑JNK, ERK1/2 ↓p38	[15]
Plant	Tomato powder			41.9 g/kg; 24 weeks	Prevention of HFD-induced inflammation Inhibition of HFD-promoted HCC development	↑SIRT1, NAMPT, NAD^+^ ↓IL1β, IL-6, IL12α, MCP1	[16]

↑, upregulation; ↓, downregulation; Bcl-2, B-cell lymphoma 2; CDK4, cyclin-dependent kinase 4; VEGF, vascular endothelial growth factor; Bax, B-cell lymphoma 2-associated X; AMPK, AMP-activated protein kinase; SIRT1, sirtuin1; LKB1, liver kinase B1; IGF-1R, insulin-like growth factor-1 receptor; PI3K, phosphatidylinositol-3-kinase; AKT, AKR mouse strain thymoma; mTOR, mammalian target of rapamycin; NF-κB, nuclear factor kappa-light-chain-enhancer of activated B cells; JNK, c-Jun-NH_2_-kinase; ERK, extracellular signal-regulated kinase; HFD, high-fat diet; HCC, hepatocellular carcinoma; NAMPT, nicotinamide phosphoribosyltransferase; NAD^+^, nicotinamide adenine dinucleotide; IL, interleukin; MCP1, monocyte chemoattractant protein-1.

**Table 2:** In vivo mice-single compounds.

Classification	Compound/Extract	Source	Experimental Model	Dose; Duration	Efficacy	Mechanism	Reference
Alkaloids	Berberine (Berberine hydrochloride)		ICR mice	50, 100 mg/kg; 15 days	Inhibition of H22 tumor growth		[17]
Alkaloids	Britanin	*Inula Linariifolia Turcz*	BALB/c mice	5, 10 mg/kg; 30 days	Inhibition of tumor proliferation Induction of apoptosis	↑Bax, ↓Bcl-2, NF-κB, p65	[18]
Fatty Acids	DOX-nano		BALB/c mice	2 mg/kg; 10 days	Inhibition of tumor growth	↓MRP, LRP, BCRP, Bcl-2, PKC-α	[19]
Fatty Acids	LDL–DHA		BALB/c mice	2.5 mg/kg; 3 days	Induction of ferroptotic cell death	↑Lipid hydroperoxides ↓GPx-4	[20]
Hydrocarbons	Atractylon	*Atractylodes lancea* *Atractylodes chinensis*	NOD/SCID mice	5, 10 mg/kg; 15 days	Inhibition of the hepatic cancer growth Inhibition of the EMT process	↑ E-cadherin, TIMP2, Bax, c- caspase-3 ↓ Ki-67, N-cadherin, Vimentin, α-SMA, MMP-2, MMP-9, Bcl-2	[21]
Hydrocarbons	GL22	*Ganoderma leucocontextum*	BALB/C nu-nu mice	50 mg/ kg; 7 days	Decrease in tumor volume	↓PPARα, PPARγ, FABP1, FABP4, FABP5	[22]
Hydrocarbons	Rotundic acid	*Ilex Rotunda*	BALB/c mice	50 mg/kg; 60 days	Inhibition of proliferation and angiogenesis Induction of apoptosis	↑p38 ↓Ki-67, CD-31	[23]
Hydrocarbons	Ursolic acid			60 mg/kg; 15 days	Inhibition of HepG2 tumor growth	↑c-caspase3 ↓p-STAT3, Bcl-2	[24]
Hydrocarbons	Zerumbone	*Zingiber zerumbet*	NSG mice	20 mg/kg; 21 days	Suppression of subcutaneous, orthotopic growth and lung metastasis		[25]
Polyphenols	747	*Abies georgei*	BALB/c mice, C57BL/6 mice	50, 100 mg/kg; 24 days	Enhancement of tumor immunosuppressive microenvironment Increase in the therapeutic effect of sorafenib	↑CD8 T cells ↓TAMs	[26]
Polyphenols	Biochanin A		Athymic nude mice	25 mg/kg; 5 weeks	Inhibition of tumor growth	↓PCNA	[27]
Polyphenols	Iscador P	*Viscum album*	Swiss Albino mice	0.1, 1, 2 mg/kg; 24, 48 h	Induction of autophagy	↑aminopeptidases, β-D-glucuronidase, hepatocyte mitochondria ↓lysosomal hydrolases	[28]
Polyphenols	*Lachnum* melanin	*Lachnum*	Kunming mice	50, 200 mg/kg; 12 days	Improvement in the immune functions Inhibition of angiogenesis	↑IL-2, IL-6, TNF-α, IFN-γ ↓VEGF, bFGF	[29]
Polyphenols	Psoralidin	*Psoralea Corylifolia*		10, 20, 40 mg/kg; 6 weeks	Inhibition of tumor growth Induction of cell cycle arrest and apoptosis	↑Bax, c-caspase-3, -9 ↓Bcl-2	[30]
Polyphenols	TTF1-NP	*Sorbaria sorbifolia*	BALB/c mice	5, 10, 20 µmol/kg	Inhibition of tumor growth	↓STAT3, p-STAT3	[31]
Polysaccharides	*Acanthopanax senticosus* extract	*Acanthopanax Senticosus*	Kunming mice	50, 100, 200 mg/kg; 10 days	Inhibition of tumor growth	↑IL-2, IL-12, INF-γ	[32]
Polysaccharides	Fucoidan		BALB/c mice	15 mg/kg, 3 weeks	Inhibition of motility and invasion of tumor	↑LINC00261 ↓miR-522-3p	[33]
Quinones	Shikonin	*Lithospermum erythrorhizon*	nu/nu mice	3 mg/kg; 30 days	Inhibition of ATO-induced tumor growth Inducement of ROS accumulation	↑CHOP, caspase 3, caspase 9, MDA	[34]

↑, upregulation; ↓, downregulation; Bcl-2, B-cell lymphoma 2; Bax, B-cell lymphoma 2-associated X; NF-κB, nuclear factor kappa-light-chain-enhancer of activated B cells; DOX-nano, doxorubicin-loaded docosahexaenoic acid nanoparticles; MRP, multidrug resistance protein; LRP, lung resistance protein; BCRP, breast cancer resistance protein; PKC-α, protein kinase C alpha; LDL–DHA, low-density lipoprotein docosahexaenoic acid; GPx-4, glutathione peroxidase-4; EMT, epithelial–mesenchymal transition; TIMP, tissue inhibitor of metalloproteinases 2; α-SMA, α-smooth muscle actin; MMP, matrix metallopeptidase; PPAR, peroxisome proliferator-activated receptor components; FABP, fatty acid-binding proteins; CD-31, cluster of differentiation 31; STAT3, signal transducer and activator of transcription 3; CD8, cluster of differentiation8; TAMs, tumor-associated macrophages; PCNA, proliferating cell nuclear antigen; IL, interleukin; TNF-α, tumor necrosis factor-α; IFN-γ, interferon-γ; VEGF, vascular endothelial growth factor; bFGF, basic fibroblast growth factor; TTF1-NP, 5,2′,4′-trihydroxy-6,7,5′-trimethoxyflavone nanoparticles; LINC00261, Long Intergenic Non-Protein Coding RNA 261; miR-522-3p, microRNA-522-3p; ATO, arsenic trioxide; ROS, reactive oxygen species; CHOP, C/EBP homologous protein.

**Table 3:** In vivo rats—mixtures.

Classification	Compound/Extract	Source	Experimental Model	Dose; Duration	Efficacy	Mechanism	Reference
Plant	*Nigella Sativa* extract	*Nigella Sativa*	Wistar Albino rats	150, 250, 350 mg/kg; 12 days	Inhibition of proliferation	↓EGFR, ERK1/2, PCNA, c-fos, Bcl-2	[39]
Plant	Ajwa dates extract	*Phoenix dactylifera*	Wistar rats	0.5, 1.0 g/kg; 10 weeks	Reversal of liver damage	↑SOD, GR, GPx, ALT, AST, ALP, IL-2, IL-12 ↓ IL-1α, IL-1β, GM-CSF, AFP, IL-6	[36]
Plant	Jianpi Huayu Decoction extract	*Panax ginseng, Atractylodes macrocephala,* *Dioscorea opposita thumb,* *Wolfiporia extens,* *Cortex Moutan Radicis,* *Salvia miltiorrhiza bunge,* *Curcuma aromatica,* *Curcuma phaeocaulis val,* *Radix Bupleuri,* *Radix liquiritiae*	Wistar rats	5 mL/dose/kg; 7 days	Inhibition of epithelial—mesenchymal transition	↑E-cadherin, EpCAM ↓N-cadherin, Vimentin	[40]
Plant	Mesohyl blue (Marine sponge extract)	*Hemimycale arabica*	Albino rats	1 mL; 4 weeks	Induction of hepatoprotective, potency, apoptosis Inhibition of proliferation	↓AFP, AFU, AST, ALT, bilirubin, CEA, GGT	[41]
Plant	Mesohyl red (Marine sponge extract)	*Negombata magnifica*	Albino rats	1 mL; 4 weeks	Induction of hepatoprotective potency, apoptosis Inhibition of proliferation	↓AFP, AFU, AST, ALT, bilirubin, CEA GGT	[41]
Plant	Purple rice bran methanol extract		Rats	500 mg/kg; 15 weeks	Inhibition of hepatocarcinogenesis	↓TNF-α, iNOS, NF- κB.	[42]

↑, upregulation; ↓, downregulation; EGFR, epidermal growth factor receptor; ERK1/2, extracellular signal-regulated kinase; PCNA, proliferating cell nuclear antigen; Bcl-2, B-cell lymphoma 2; SOD, superoxide dismutase; GR, glutathione reductase; GPx, glutathione peroxidase; ALT, alanine aminotransferase; AST, aspartate aminotransferase; ALP, alkaline phosphatase; IL, interleukin; GM-CSF, granulocyte-macrophage colony-stimulating factor; AFP, alpha-feto protein; EpCAM, epithelial cell adhesion molecule; AFU, alpha-fucosidase; CEA, carcinoembryonic antigen; GGT, gamma glutamyl peptidase; TNF-α, tumor necrosis factor-α; iNOS, inducible nitric oxide synthase; NF-κB, nuclear factor-κB.

**Table 4:** In vivo rats—single compounds.

Classification	Compound/Extract	Source	Experimental Model	Dose; Duration	Efficacy	Mechanism	Reference
Fatty Acids	LDL–DHA		AxC-Irish rats	2 mg/kg	Inhibition of redox reactions in tumor tissues	↓GPx-4	[43]
Hydrocarbons	Phyllanthin		Wistar Albino rats	30 mg/kg; 14 weeks	Inhibition of tumorigenesis Suppression of liver damage	↑p53, Bax, c-caspase-3,9 ↓Bcl-2	[44]
Hydrocarbons	Zerumbone	*Zingiber zerumbet*	Sprague Dawley rats	30, 60 mg/kg; 3 weeks	Induction of apoptosis Inhibition of angiogenesis	↓Ki-67, VEGF, MMP-9	[38]
Polysaccharides	Arabinoxylan	*Oryza Sativa*	Wistar rats	25 mg/kg; 22 weeks	Induction of cell cycle arrest, DNA fragmentation in cancer cells, apoptosis Inhibition of hepatocarcinogenesis	↑Bax, c-caspase-3, p53, IκB-α, NF-κB/p65 ↓Bcl-2	[45]
Polyphenols	Dieckol	*Ecklonia cava*	Wistar rats	40 mg/kg; 15 weeks	Regulation of apoptosis, inflammation, invasion, and angiogenesis	↓XMEs, Bcl-2, MMP2/9, VEGF, NF- κB, COX2 ↑Bax, cytochrome c, caspase-3	[46]
Polyphenols	Hesperetin		Wistar rats	20 mg/kg; 16 weeks	Inhibition of cell inflammation and proliferation	↓TNF-α, NF-κB, glycoconjugates, PCNA	[47]
Quinones	Coenzyme Q10		Albino rats	0.4 mg/kg; 2 weeks	Inhibition of proliferation, histological alterations	↑c-caspase-3 ↓CD59, Bcl-2, SRB1, PLD	[48]

↑, upregulation; ↓, downregulation; LDL–DHA, low-density lipoprotein docosahexaenoic acid; GPx-4, glutathione peroxidase-4; Bax, B-cell lymphoma 2-associated X; Bcl-2, B-cell lymphoma 2; VEGF, vascular endothelial growth factor; MMP, matrix metalloproteinases; IκB-α, nuclear factor of kappa light polypeptide gene enhancer in B-cells inhibitor α; NF-κB, nuclear factor-κB; XMEs, xenobiotic-metabolizing enzymes; COX2, cyclooxygenase2; TNF-α, tumor necrosis factor-α; PCNA, proliferating cell nuclear antigen; CD59, cluster of differentiation 31; SRB1 scavenger receptor class B type 1; PLD, phospholipase D.

**Table 5:** Chemical structures of the single compounds.

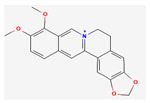 Berberine	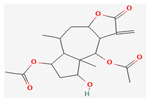 Britanin	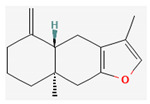 Atractylon	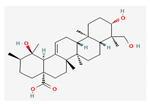 Rotundic acid	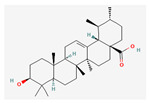 Ursolic acid
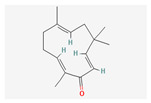 Zerumbone	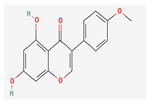 Biochanin A	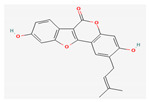 Psoralidin	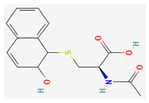 *Acanthopanax senticosus* extract	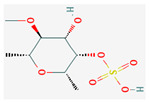 Fucoidan
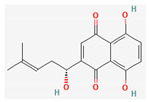 Shikonin	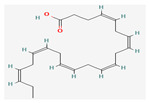 LDL–DHA	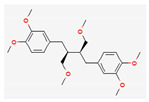 Phyllanthin	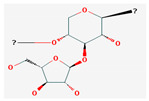 Arabinoxylan	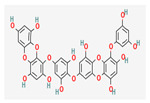 Dieckol
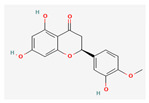 Hesperetin	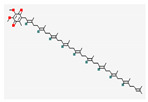 Coenzyme Q10			

**Table 6:** Clinical trials.

Compound/Extract	Source	Patients	Efficacy	Results of the Treatment Groups	Results of the Control Groups	*p*-Value	Registration Number	Reference
Fuzheng Jiedu Xiaoji formulation	*Codonopsis pilosula* *Astragalus mongolicus* *Atractylodes macrocephala* *Angelica sinensis* *Poria cocos* *Adenophora stricta* *Ophiopogon japonicus* *Rehmannia glutinosa* *Paris polyphylla* *Curcuma phaeocaulis* *Pinellia ternata*	291	Inhibition of the proliferation and migration of liver cancer cells Extension of one-year OS and PFS	One-year OS rate was 80.6%, and PFS rate was 48.6%	One-year OS rate was 68%, and PFS rate was 27.9%	0.0233 (OS) 0.0064 (PFS)		[50]
Jiedu Granule	*Salvia chinensis* *Actinidia valvata* *Gallus gallus domesticus* *Cremastra appendiculata*	190	Extension of median overall survival rates	The median OS was 15.8 months	The median OS was 11.3 months	0.00047		[51]
PHY906	*Scutelleria baicalensis* *Paeonia lactiflora* *Glycyrrhiza uralensis* *Ziziphus jujuba*	39	Extension of median overall survival rates	The median PFS was 1.5 months, and median OS was 6 months with a 51.3% 6-month survival rate.	N/A	N/A	NCT00076609	[52]
THM	*Bufo gargarizans* *Actinidia valvata* *Salvia chinensis* *Pseudobulbus cremastrae* *Gallus gallus domesticus*	364	Prevention of small hepatocellular carcinoma recurrence	The median RFS was 85.83 months and the 5-year OS rates was 71.11%	The median RFS was 26.00 months and the 5-year OS rates was 63.04%	<0.001 (RFS) 0.0076 (OS)		[53]
Huaier granule	*Trametes robiniophila*	62	Improvement in treatment response Extension of median survival rates	The 6- and 12-month OS were 100% and 93.5%, respectively	The 6- and 12-month OS were 90.3% and 80.6%, respectively	<0.05 (6 month) >0.05 (12 month)		[54]
Jian Pi Li Qi decoction	*Poria cocos,* *Atractylodes macrocephala* *Codonopsis pilosula* *Fructus aurantii* *Akebia fruit* *Citrus Chirocarpus*	140	Improvement in liver function	N/A	N/A	N/A		[55]
Shuangbai San	*Radix et Rhizoma Rhei* *Platycladus orientalis* *Phellodendron amurense* *Lycopus lucidus* *M. haplocalyx*	134	Relief of cancer pain and improvement in quality of life	N/A	N/A	N/A		[56]
Yunzhi	*Coriolus Versicolor*	15	Extension of median survival rates	The median PFS was 2.5 months, and the median OS was 6.5 months	The median PFS was 1.1 months, and the median OS was 2.2 months	0.144 (PFS) 0.105 (OS)		[57]

OS, overall survival; PFS, progression-free survival; THM, Traditional herbal medicine; RFS, recurrence-free survival.
